# Synthesis, in vitro and in vivo evaluation of ^11^C-*O*-methylated arylpiperazines as potential serotonin 1A (5-HT_1A_) receptor antagonist radiotracers

**DOI:** 10.1186/s41181-020-00096-8

**Published:** 2020-05-19

**Authors:** Vidya Narayanaswami, Junchao Tong, Ferdinando Fiorino, Beatrice Severino, Rosa Sparaco, Elisa Magli, Flavia Giordano, Peter M. Bloomfield, Jaya Prabhakaran, J. John Mann, Neil Vasdev, Kenneth Dahl, J. S. Dileep Kumar

**Affiliations:** 1 Azrieli Centre for Neuro-Radiochemistry, Research Imaging Centre & Preclinical Imaging, Centre for Addiction and Mental Health, Toronto, Ontario M5T-1R8 Canada; 2 Department of Pharmacy, University of Naples, Via D. Montesano, 49, 8013 Naples, Italy; 3 Molecular Imaging and Neuropathology Division, New York State Psychiatric Institute, New York, USA; 4 Department of Psychiatry, Columbia University Medical Center, New York, USA; 5 Department of Psychiatry, University of Toronto, Toronto, Ontario M5T-1R8 Canada

**Keywords:** α_1_-adrenergic receptor, Carbon-11, 5-HT_1A_ receptor, Serotonin, PET

## Abstract

**Background:**

Serotonin 1A (5-HT_1A_) receptors are implicated in the pathogenesis of several psychiatric and neurodegenerative disorders motivating the development of suitable radiotracers for in vivo positron emission tomography (PET) neuroimaging. The gold standard PET imaging agent for this target is [*carbonyl*-^11^C]WAY-100635, labeled via a technically challenging multi-step reaction that has limited its widespread use. While several antagonist and agonist-based PET radiotracers for 5-HT _1A_ receptors have been developed, their clinical translation has been hindered by methodological challenges and/or and non-specific binding. As a result, there is continued interest in the development of new and more selective 5-HT_1A_ PET tracers having a relatively easier and reliable radiosynthesis process for routine production and with favorable metabolism to facilitate tracer-kinetic modeling. The purpose of the current study was to develop and characterize a radioligand with suitable characteristics for imaging 5-HT_1A_ receptors in the brain. The current study reports the in vitro characterization and radiosyntheses of three candidate 5-HT_1A_ receptor antagonists, DF-100 **(1)**, DF-300 **(2)** and DF-400 **(3)**, to explore their suitability as potential PET radiotracers.

**Results:**

Syntheses of **1–3** and corresponding precursors for radiolabeling were achieved from isonicotinic, picolinic acid or picolino nitrile. In vitro binding studies demonstrated nanomolar affinity of the compounds for 5-HT_1A_ receptors. Binding of **1–3** for other biogenic amines, neurotransmitter receptors, and transporters was negligible with the exception of moderate affinities for α_1_-adrenergic receptors (4–6-fold less potent than that for 5-HT_1A_ receptor). Radioligands [^11^C]**1**–**3** were efficiently prepared by ^11^C-*O*-methylation of the corresponding phenolic precursor in non-decay corrected radiochemical yields of 7–11% with > 99% chemical and radiochemical purities. Dynamic PET studies in rats demonstrated negligible brain uptake of [^11^C]**1** and [^11^C]**2**. In contrast, significant brain uptake of [^11^C]**3** was observed with an early peak SUV of 4–5. However, [^11^C]**3** displayed significant off-target binding attributed to α_1_-adrenergic receptors based on regional distribution (thalamus>hippocampus) and blocking studies.

**Conclusion:**

Despite efficient radiolabeling, results from PET imaging experiments limit the application of [^11^C]**3** for in vivo quantification of 5-HT_1A_ receptors. Nevertheless, derivatives of compound 3 may provide a scaffold for alternative PET radiotracers with improved selectivity for 5-HT _1A_ receptors or α_1_-adrenergic receptors.

## Introduction

5-Hydroxytryptamine or serotonin (5-HT) is a major neurotransmitter and neuromodulator at central and peripheral sites (Barnes and Sharp 1999). Physiologically, 5-HT is crucial in the control of sleep, wakefulness, mood, feeding behavior, learning and memory, decision-making and the control of sensory transmission. Disruption in 5-HT neurotransmission and/or 5-HT receptor function has been implicated in the pathophysiology of several neuropsychiatric and neurodegenerative disorders that include: major depression, anxiety disorders, schizophrenia, sleep disorders, Alzheimer’s disease, Parkinson’s disease and epilepsy (Burnet et al. 1997; Merlet et al. 2004; Sullivan et al. 2005; Schmitt et al. 2006; Kepe et al. 2006; Ballanger et al., 2012; Michelsen et al. 2008; Pagano and Politis 2018). To date, fourteen 5-HT receptor subtypes have been identified and have been divided into seven classes, 5-HT_1_ to 5-HT_7_, according to their structural and functional characteristics. The 5-HT_1A_ subtype is amongst the best characterized. The 5-HT_1A_ receptor is a G-protein-coupled receptor concentrated in cortical and limbic regions that receive serotonergic input from the raphe nuclei such as the frontal cortex, amygdala, and hippocampus (Weissmann-Nanopoulos et al. 1985; Hoyer et al. 1986; Radja et al. 1992). The 5-HT_1A_ receptor serves predominantly as an autoreceptor that controls 5-HT release from serotonin neurons in the raphe nuclei by hyper-polarizing the neuron after serotonin release from recurrent short fibers that terminate on the soma and dendrites of serotonin neurons. Thus, this receptor regulates 5-HT neurotransmission to its projection areas, and is expressed by target neurons as a postsynaptic receptor in frontal and limbic projection regions (Weissmann-Nanopoulos et al. 1985; Radja et al. 1992).

Clinical relevance of 5-HT_1A_ receptors in the pathogenesis of several psychiatric and neurodegenerative disorders has encouraged significant efforts in developing both carbon-11 and fluorine-18 labeled radiotracers for in vivo positron emission tomography (PET) neuroimaging studies to investigate alterations of 5-HT_1A_ receptors in human brain. These imaging approaches may not only provide insight into disease diagnosis, subtypes and its progression, but also provide a biomarker of disease response, and permit receptor occupancy studies of drugs. Over the past three decades, several 5-HT_1A_ receptor antagonist- and agonist-based PET or SPECT radioligands have been evaluated for imaging purposes (Kumar and Mann 2014). Currently available PET radiotracers for 5-HT_1A_ exhibit structural similarity to the 5-HT_1A_ antagonist, *N*-[2-[4-(2-methoxyphenyl)-1-piperazinyl]ethyl]-*N*-(2-pyridinyl) cyclohexane carboxamide (WAY100635). At present, [*carbonyl*-^11^C]WAY100635 (Osman et al. 1996; Krasikova et al. 2009), [^11^C]DWAY (Pike et al. 1998; Andree et al. 2002), [^18^F]FCWAY (Choi et al. 2014), and [^18^F]MPPF (Shiue et al. 1997) are the reported antagonist PET ligands for the quantification of 5-HT_1A_ receptors in humans. [*carbonyl*-^11^C]WAY100635, with significant improvements over its predecessor, [*O-methyl*-^11^C]WAY100635, on brain-penetrating metabolites (Osman et al. 1996), is still the gold standard radiotracer for 5-HT_1A_ brain imaging in humans and has the highest specific to non-specific binding ratios among the 5-HT_1A_ tracers. There are discrepant reports about the non-displaceable binding potential (BP) of [*carbonyl*-^11^C]WAY100635 in patients with major depressive disorder as only marginal non-specific binding is observed in the cerebellar vermis (Drevets et al. 1999; Sargent et al. 2000; Meltzer et al. 2004; Parsey et al. 2006; Hirvonen et al. 2008). Due to the low non-specific binding in cerebellum, the BP, based on measurement of the free fraction of radioligand in plasma, can only be accurately obtained with full arterial input function and can lead to variability in binding outcome measurements. The challenging kinetic measurements of [*carbonyl*-^11^C]WAY100635 coupled with rapid metabolism, low free fraction, complicated radiosynthesis and low yield, and the short half-life of carbon-11 have been ongoing motivations to develop alternative PET radiotracers for imaging the 5-HT_1A_ receptors.

[^18^F]FCWAY, the fluoro-analogue of WAY100635 has been tested as an alternative; however, potential in vivo defluorination is the major drawback of this tracer (Choi et al. 2014). While [^18^F]MPPF, the fluorophenyl analogue of WAY100635, exhibited optimum sensitivity to measure intra-synaptic 5-HT levels in vivo in rodents, studies in awake monkeys and human subjects did not show such effect. Moreover, [^18^F]MPPF is a P-glycoprotein substrate that limits further utility in clinical application (Shiue et al. 1997; Aznavour and Zimmer 2007). In addition to nanomolar affinity of [^11^C](*R*)-RWAY, the reverse amide of WAY100635, to 5-HT_1A_ receptors (Ki = 0.6 nM), this radiotracer also possesses significant affinity to 5-HT_2B_ receptors (Ki = 7.2 nM), alpha-1 (α_1_) adrenergic receptors (Ki = 10.35 nM), and dopaminergic receptors (D2, Ki = 34.5 nM; D3, Ki = 5.1 nM; and D4, Ki = 15.6 nM) (Yasuno et al. 2006). Moreover, the presence of radioactive metabolites in the brain and its binding to P-glycoprotein restricts its clinical translation (Zhang et al. 2007). [^18^F](trans)-MeFWAY has been successfully evaluated in human subjects (Mukherjee et al. 2016). However, kinetic analyses with arterial input functions have to be performed for the full quantification of this radiotracer.

Development of agonist-based 5-HT_1A_ receptors has also faced certain challenges primarily due to a lack of detectable specific binding. Among these, [^11^C]CUMI-101 or [^11^C]MMP have been investigated in non-human primates and human subjects (Kumar et al. 2007; Milak et al. 2010). Pre-treatment with the α_1_-adrenergic receptor antagonist, prazosin, demonstrated partial displacement of [^11^C]CUMI-101 binding in the thalamus and cerebellum of rats and monkeys indicating moderate affinity of CUMI-101 to α_1_-adrenergic receptor (Shrestha et al. 2014). However, such effect was not found in in vitro autoradiography studies in non-human primate and human brain sections with [^3^H]CUMI-101 (Kumar et al. 2013). On the other hand, despite specific in vitro binding of [^11^C]MPT in 5-HT_1A_ receptor-rich regions, the slow washout in baboons complicates the quantification of binding parameters (Kumar et al. 2006).

Given the aforementioned limitations that restrict the clinical utility of currently available 5-HT_1A_ receptor radiotracers, there is continued interest in the development of new and more selective 5-HT_1A_ PET tracers having a relatively easier and reliable radiosynthesis process for routine production and with favorable metabolism in order to facilitate tracer-kinetic modeling. To that end, the purpose of the current study was to develop and characterize a radioligand with suitable characteristics for imaging 5-HT_1A_ receptors in the brain. Most 5-HT_1A_ receptor PET ligands developed to date are analogues of WAY100635 or the arylpiperazine scaffold and have been achieved with limited success (Kumar and Mann 2014). We sought to develop PET tracers of WAY100635 analogues without the cyclohexane group in view of enhancing 5-HT_1A_ receptor selectivity and improving brain uptake and non-specific binding by lowering the lipophilicity. Among the reported arylpiperazine analogues; isonicotinamide, picolinamide bearing a 2-carbon (ethyl) linker to the amide with a *O*-methoxy phenyl group and, *N*-cyanonicotimamide bearing three carbon (propyl) linkers showed high affinity to 5-HT_1A_ and 5-HT_2A_ receptors (Fiorino et al. 2012; Fiorino et al. 2016; Fiorino et al. 2017). The binding assays for these ligands were performed using 3 concentrations with hill co-efficients less than one; however, receptor selectivity data has not been previously reported. We herein present the detailed in vitro binding characterization of these three high affinity 5-HT_1A_ receptor antagonists amenable for radiolabeling by standard ^11^C-*O*-methylation reactions. We further explore the feasibility of the resultant ^11^C-labeled radioligands to image 5-HT_1A_ receptors by conducting preliminary PET/MR imaging in rodents.

## Methods

### Chemistry and in vitro pharmacological characterization of novel 5-HT_1A_ receptor ligands; DF-100 (1), DF-300 (2) and DF-400 (3)

Syntheses of compounds **1–3** were achieved using previously established procedures (Fiorino et al. 2012, Fiorino et al. 2016, Fiorino et al. 2017). The synthesis of desmethyl-DF-100, the radiolabeling precursor, was achieved from 2-cyanopyridine in three steps. Methyl-*N*-cyano-2-pyridinecarboximidate obtained by reacting 2-cyanopyridine with cyanamide was coupled with 3-bromopropylamine and subsequent condensation of resulting cyanopicolinamidine with 2-hydroxyphenylpiperazine afforded desmethyl-DF-100. Synthesis of desmethyl-DF-300 and desmethyl-DF-400 were achieved from picolinic acid or isonicotinic acid by reacting with 2-chloroethanamine followed by condensation with 2-hydroxyphenyl-piperazine. Details of the chemical syntheses scheme is provided in the supplementary information.

Binding affinity (K_i_) of compounds **1**–**3** at 5-HT_1A_ receptors were determined by competition binding studies with [^3^H]WAY100635 employing 12 concentrations of the compounds (10 μM to 1 pM) in triplicate measurements and using 8-hydroxy-2-(di- n-propilamino)tetralin (8-OH-DPAT, a 5-HT_1A_ agonist) as a reference standard in stable chicken hamster ovary cells expressing 5-HT_1A_ receptor (National Institute of Mental Health-Psychoactive Drug Screening Program (NIMH-Psychoactive Drug Screening Program (PDSP)). Cross selectivity for biogenic amines, neurotransmitter receptors, and transporters were determined by radioligand binding assays through NIMH- PDSP using validated and established protocols (NIMH-PDSP Assay Protocol Book Version III, March 2018).

### Radiosyntheses

Details pertaining to the syntheses of radiolabeling precursors for **1–3** are provided in the supplementary information. Unless otherwise stated, all reagents and solvents used for radiosynthesis were purchased from Sigma Aldrich (St. Louis, Missouri, US) and used without further purification. Quality control high performance liquid chromatography (HPLC) analysis was performed using a high-pressure isocratic pump (LC-20AT; Shimadzu Inc., Kyoto, Japan) and a variable wavelength ultraviolet (UV) detector (λ = 254 nm, SPD-20A, Shimadzu Inc.) in a series with a radioactivity detector (Frisk-tech, Bicron; Torrington, Connecticut, US) connected in series. The system was equipped with a reverse phase analytical HPLC (Luna C-18, 10 μm, 4.6 × 250 mm, Phenomenex; Torrance, California, US) and controlled by PowerChrom chromatography software (eDAQ Pty Ltd.; Colorado Springs, Colorado, US).

*Preparation of [*^*11*^*C]methyl iodide ([*^*11*^*C]CH*_*3*_*I):* [^11^C]CH_3_I was produced using a previously reported gas-phase iodination method (Larsen et al. 1997). No-carrier-added [^11^C]carbon dioxide ([^11^C]CO_2_) production was performed using a MC17 cyclotron (Scanditronix; Uppsala, Sweden). The ^14^N(p, α)^11^C reaction was employed in a pressurized gas target containing nitrogen and 0.5% oxygen by bombardment with 30 μA proton beam for 30 min (∼37 GBq of [^11^C]CO_2_). [^11^C]CO_2_ was delivered from the cyclotron target via a 1/8″ stainless-steel delivery line by nitrogen pressure directly to a column packed with 0.3 g of molecular sieve and 0.2 g of nickel (Shimalite-Ni (reduced), Shimadzu Inc.) where it was trapped at room temperature. The column was then sealed under hydrogen gas and heated to 350 °C for 60 s to reduce the [^11^C]CO_2_ to [^11^C]CH_4_. The [^11^C]CH_4_ was passed through a column of phosphorus pentoxide and trapped on a column of carbosphere cooled to − 75 °C (with liquid nitrogen). Gaseous [^11^C]CH_4_ was released by heating the carbosphere column to 80 °C. Once released, the [^11^C]CH_4_ entered a circulation loop, which includes a membrane-based gas pump, a column of iodine at 100 °C, a quartz-glass iodine reactor tube at 740 °C, two adjacent columns of Ascarite, and a column of Porapak Q at room temperature. The gaseous mixture was circulated for 5 min, whereas [^11^C]CH_3_I accumulated on the Porapak column. [^11^C]CH_3_I (15 GBq, 400 mCi) was then released from the Porapak column and delivered directly to the reaction vessel using a control stream of He flow (10 mL/ min) while heating the Porapak column to 190 °C.

*Synthesis of [*^*11*^*C]DF-100 ([*^*11*^*C]****1****), [*^*11*^*C]DF-300 ([*^*11*^*C]****2****) and [*^*11*^*C]DF-400 ([*^*11*^*C]****3****):* [^11^C]CH_3_I was trapped in the reaction mixture containing the corresponding radiolabeling precursor at room temperature. Reaction mixture for respective radiotracer synthesis were as follows: [^11^C]**1**: 0.5 mg of desmethyl-DF-100, 3 μL TBAOH (1 M in MeOH), 300 μL DMSO; [^11^C]**2**: 0.5 mg of desmethyl-DF-300, 3 μL NaOH (1 M in H_2_O), 300 μL DMF; [^11^C]**3**: 0.5 mg of desmethyl-DF-400, 3 μL NaOH (1 M in H_2_O), 300 μL DMF. After the end of radioactivity delivery, the reaction vial was heated at 70 °C for 3 min. The reaction was quenched with 1.0 mL of water and injected onto a HPLC column (Nucleosil C-18 Nautilus. 5 μm, 10 × 250 mm, MACHEREY-NAGEL GmbH & Co; Düren, Germany) for further purification. Radioligands were eluted with following mobile phase composition: [^11^C]**1** and [^11^C]**3**: 25:75 CH_3_CN/0.1 N ammonium formate; [^11^C]**2**: 30:70 CH_3_CN/0.1 N ammonium formate. All three radiotracers were eluted with a flowrate of 5 mL/min. The eluent was monitored by UV (λ = 254 nm) and radioactivity detectors connected in series (R_t_ [^11^C]**1** = 13 min; R_t_ [^11^C]**2** = 12 min; R_t_ [^11^C]**3** = 13.5 min). The product was diluted with 25 mL of sterile water. The diluted HPLC fraction was then loaded on a solid-phase extraction cartridge (SepPak tC18 Plus, Waters; Milford, Massachusetts, US), then washed with 10 mL of sterile water. Radiotracers were recovered in 1.0 mL of dehydrated ethanol for injection, USP, and 10 mL of 0.9% sodium chloride for injection, USP. [^11^C]**1** was obtained in 9% radiochemical yield (RCY) (non-decay corrected) at end-of-synthesis (39 min) based upon [^11^C]CO_2_, with > 99% radiochemical purity (RCP) and in a molar activity (A_m_) of 99 GBq/μmol. [^11^C]**2** was obtained in 7% RCY (non-decay corrected) at end-of-synthesis (37 min) based upon [^11^C]CO_2_, with > 99% RCP and in a A_m_ of 81 GBq/μmol. [^11^C]**3** was obtained in 7.5 ± 1.5% (*n* = 5) RCY (non-decay corrected) at end-of-synthesis (38 min) based upon [^11^C]CO_2_, with > 99% RCP and in a A_m_ of 131 ± 32 GBq/μmol. Product identity and purity were determined by radio-HPLC (30:70 CH_3_CN/0.1 N ammonium formate) and UV by co-injection with the standard.

### In vivo small animal PET/MR imaging study

All three candidate 5-HT_1A_ radioligands, [^11^C]**1**, [^11^C]**2**, and [^11^C]**3**, were tested in dynamic PET studies in rats to further investigate blood-brain barrier (BBB) permeability, regional brain distribution, and tracer kinetics. All experimental procedures were carried out in accordance with the Institutional Animal Care Committee ethical guidelines (Animal Use Protocol # 783).

#### Animal preparation

Adult male Sprague Dawley rats (500–600 g, 8–12 months old) were anesthetized by isoflurane (5% induction; O_2_ rate: 2 L/min) and catheterized in the lateral tail vein using a Surflash Polyurethane IV Catheter 24G × 3/4″(Terumo; Somerset, New Jersey, US). Following insertion, the catheter was flushed with heparinized saline (30 IU/ml, ~ 200 μl). The animal was transferred to the scanner bed in prone position, the head immobilized in a flat skull position for the duration of the acquisition using built in ear and bite bars; the scanner bed temperature was initially set at 40 °C but was subject to alteration based on animals’ body temperature during the experiment. Anesthesia was maintained throughout the PET/MR scanning procedure (isoflurane: 1.5–2%; O_2_ rate: 1 L/min) and the animals’ body temperature and respiration parameters were closely monitored.

#### PET/MR acquisition

Imaging studies were conducted on a nanoScan PET/MRI 3 T tomograph (Mediso; Budapest, Hungary). At first, a scout MR was acquired for subsequent PET field of view (FOV) positioning. MR images were used to define anatomical regions of interest (ROIs) through PET/MRI image co-registration. MR sequences included: material map T1-weighted 2D-gradient echo (GRE, TR 354 ms, TE 3.64) multi-FOV sequence for PET and MR co-registration and PET scatter and attenuation corrections, and T2-weighted 2D-fast spin-echo (FSE, TR 3971 ms, TE 87.5 ms) sequence for co-registration with standard rat brain MR template and atlas of Schwarz et al. (2006), and PET ROI analyses. MR images were acquired either before or after PET imaging.

Three separate imaging experiments were conducted to determine the BBB permeability of [^11^C]**1**, [^11^C]**2**, and [^11^C]**3**, respectively. Concomitantly with a bolus injection of each individual radiotracer (injected radioactivity range for [^11^C]**1–3**: 14–22 MBq (molar activities at the time of injection for [^11^C]**1–3**: 75–180 GBq/μmol; mass injected for [^11^C]**1–3**: 0.15–0.40 nmol/kg), a 60 min emission list mode scan was acquired with an energy window of 400–600 keV.

5-HT_1A_ receptor selectivity of the radiotracer with the most promising brain penetrating properties was further investigated in two separate baseline and pre-treatment (blocking) experiments. A within-subject PET/MR imaging design was employed for baseline and blocking experiments. In the first PET/MR imaging session, brain radiotracer uptake was determined under baseline conditions whereby radiotracer was intravenously administered simultaneously with the start of PET acquisition. The catheter was flushed with heparinized saline (~ 100 μl) and capped to allow blocker/radiotracer injections for the subsequent pre-treatment experiment. Before the start of the second PET measurement, the animals were infused with a suitable blocking agent intravenously 20 min prior to radiotracer injection and concomitant PET acquisition. To confirm target engagement at 5-HT_1A_ receptor, WAY-100635 maleate (Sigma-Aldrich), a potent 5-HT_1A_ receptor antagonist (2 mg/kg) was infused intravenously 20 min before radiotracer injection. In order to further investigate potential off-target binding at α_1_-adrenergic receptors in vivo, prazosin hydrochloride (Sigma-Aldrich), a potent α_1_-adrenergic receptor antagonist (2 mg/kg) was infused 20 min before radiotracer injection and PET acquisition.

#### PET data analyses

The acquired list mode data was sorted into 33, 3D (3 × 5 s, 3 × 15 s, 3 × 20s, 7 × 60s and 17 × 180 s) true sinograms (ring difference 84). The 3D sinograms were converted in 2D sinograms using fourier-rebinning (Defrise et al. 1997), during which corrections were included for detector geometry and efficiencies, attenuation and scatter, prior to image reconstruction using a 2D–filtered back projection (FBKP) with an Hann filter at a cutoff of 0.50 cm-1. A static image of the complete emission acquisition was reconstructed with the manufacturer’s proprietary iterative 3D algorithm (6 subsets, 4 iterations). All image data were corrected for dead-time and decay corrected to the start of acquisition. The dynamic FBKP images were used for the extraction of time-activity curves (TACs). The static iterative image was used for PET and MR co-registration and also for co-registration with standard rat brain MR template and atlas (Schwarz et al. 2006). Image analyses and extraction of regional brain TACs were performed using Amide software (v1.0.4) (Loening and Gambhir 2003). Standardized uptake values (SUV) were calculated by normalizing regional radioactivity for injected radioactivity and body weight of the animal. Regional brain BP were obtained by using simplified reference tissue model (SRTM) of Lammertsma and Hume (1996), with averaged TACs of left and right cerebellum as the reference. This was performed by using the basis function method (BFM) of Gunn et al. (1997) in the package of Turku PET Centre (https://gitlab.utu.fi/vesoik/tpcclib) (Gunn et al. 1997). Percentages of blocking were calculated by the equation: Blocking% = 100 x (BP_blocking_ – BP_baseline_)/BP_baseline_.

## Results and discussion

In the current study, we report the in vitro pharmacological characterization, radiosyntheses and preliminary in vivo PET imaging of three new 5-HT_1A_ receptor arylpiperazine based ligands in rats.

### In vitro pharmacological characterization

**Fig. 1:**
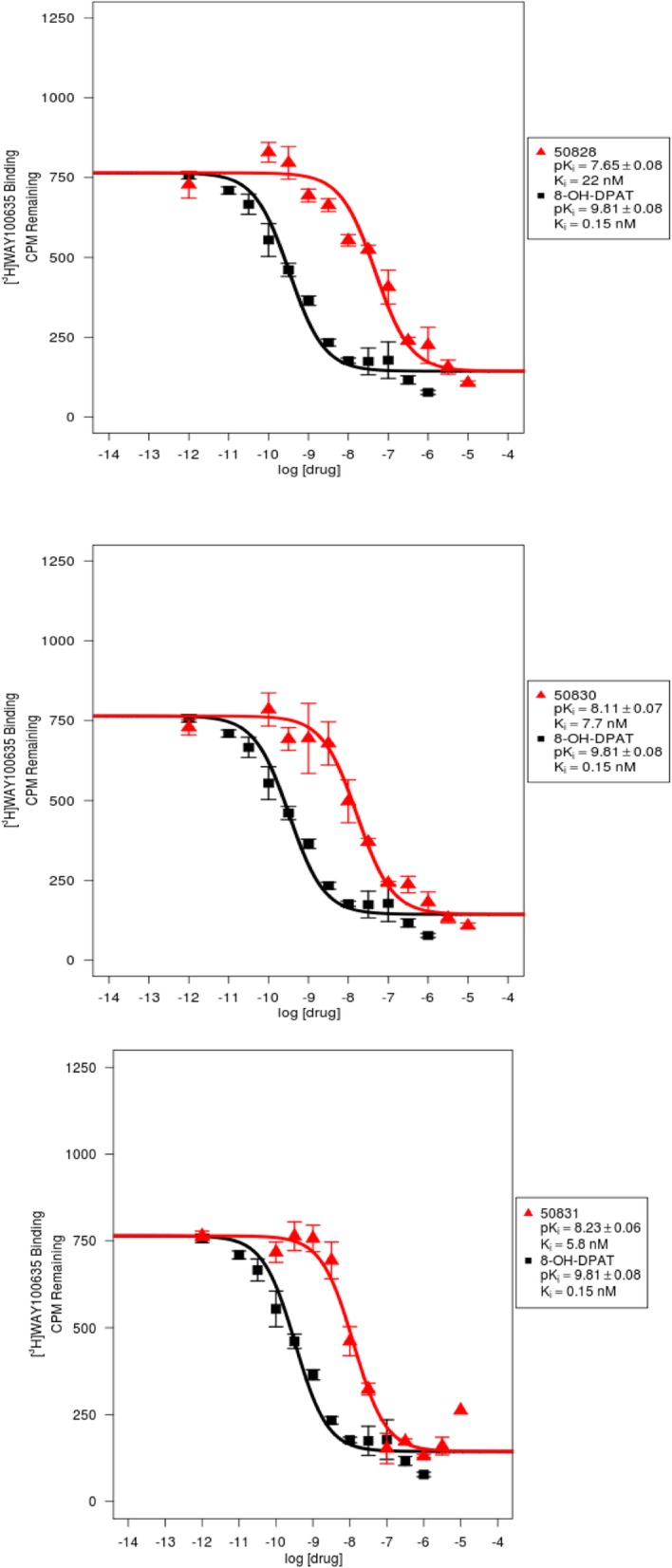
Binding affinity (Ki) of compounds 1–3 at 5-HT_1A_ receptors: Competition binding curves for DF-100 (1) (PDSP#50828, top), DF-300 (2) (PDSP#50830, middle) and DF-400 (3) (PDSP#50831, bottom) in the presence of [^3^H]WAY100635 and 8-OH-DPAT as reference standard

Results demonstrated nanomolar affinity (K_i_) of the candidate ligands to 5-HT_1A_ receptors (**1**: 22 nM, **2**: 7.7 nM and **3**: 5.8 nM) (Fig. 1) and α_1_-adrenergic receptors (**1**: 129 nM, **2**: 46 nM and **3**: 23 nM) (Table 1). In vitro data indicate relatively higher binding affinity of compound **3** for 5-HT_1A_ receptors compared to other candidate 5-HT_1A_ receptor ligands and ~ 4-fold selectivity to 5-HT_1A_ receptors over α_1_-adrenergic receptors. Affinity of the candidate ligands for other biogenic amines, receptors, and transporters were low (K_i_ = 0.1 to > 10 μM) (Table 1). Binding affinity ratios of 5-HT_1A_ and α_1_-adrenergic receptors are ~ 6 for compounds **1** and **2** and 4 for compound **3**. We did not perform saturation binding studies to determine B_max_ and K_d_ of compounds **1–3** for 5-HT_1A_ and α_1_-adrenergic receptors. Both 5-HT_1A_ and α_1_-adrenergic receptors are highly abundant in hippocampus and cortical brain regions (B_max_: 100–200 fmol/mg/protein), whereas, 5-HT_1A_ receptors are less expressed in cerebellum, thalamus and striatum (4–10 fmol/mg/protein) compared to α_1_-adrenergic receptors (40–100 fmol/mg/protein) (Burnet et al. 1997; Hall et al. 1997; Kalaria 1989; Khawaja 1995; Kumar et al. 2013; Shimohama et al. 1986). Therefore, given the 4–6-fold high affinity ratios of 5-HT_1A_ vs. α_1_-adrenergic receptors, we expected the ^11^C-labeled compounds **1–3** to bind preferentially to 5-HT_1A_ receptor site. It should be indicated that previous preliminary pharmacological screening of the current candidate ligands has reported high affinities to other serotonin receptors including 5-HT_2A_ (compounds **1** and **2**) and 5-HT_2C_ (compounds **1** and **3**) (Fiorino et al. 2012; Fiorino et al. 2016; Fiorino et al. 2017). Compounds **1** and **3** also exhibit high affinity to 5-HT_1A_ receptors (K_i_ = 4.68 nM for compound **1** and K_i_ = 0.36 nM for compound **3**) (Fiorino et al. 2012, Fiorino et al. 2016, Fiorino et al. 2017). Discrepancies between previous reports and the current study are attributed to differences in the binding assay methodology. Previous reports do not provide binding curves for compounds **2** and **3**. Also, the curve for compound **1** was derived using only 3-concentrations (1 nM, 10 pM and 0.1 pM) and the reference compound and standard deviation were not stated. Herein, we report results from competitive binding assays of all the three compounds obtained via the NIMH-PDSP and confirm that the ligands have negligible affinity to 5-HT_2_ receptors (Table 1).

**Table 1:** Binding affinity (K_i_) of compounds **1–3** for 5-HT_1A_ receptors and other biogenic amines, neurotransmitter receptors, and transporters

	(1)	(2)	(3)
**Target**	**K**_**i**_**(nM)**	**K**_**i**_**(nM)**	**K**_**i**_**(nM)**
**5-HT**_**1A**_	**22**	**7.7**	**5.8**
5-HT1B	> 10,000	> 10,000	> 10,000
5-HT1E	> 10,000	> 10,000	> 10,000
5-HT1D	> 10,000	> 10,000	1176
5-HT2A	> 10,000	> 10,000	> 10,000
5-HT2B	403	516	137
5-HT2C	> 10,000	> 10,000	> 10,000
5-HT3	> 10,000	> 10,000	> 10,000
5-HT4	> 10,000	> 10,000	> 10,000
5-HT5a	> 10,000	> 10,000	1805
5-HT6	> 10,000	> 10,000	> 10,000
5-HT7R	76	830	530
**Alpha1A**	**129**	**46**	**23**
Alpha1B	1080	254	142
Alpha1D	213	53	19
Alpha 2A	453	> 10,000	> 10,000
Alpha2B	670	> 10,000	2215
Alpha2C	835	> 10,000	1984
D2R	777	> 10,000	800
D3R	369	2552	133
D4R	137	88	153
D1, D5	> 10,000	> 10,000	> 10,000
DAT	> 10,000	> 10,000	> 10,000
NET	> 10,000	> 10,000	> 10,000
SERT	> 10,000	> 10,000	> 10,000
Adenosine receptors	> 10,000	> 10,000	> 10,000
AMPA	> 10,000	> 10,000	> 10,000
Beta Receptors	> 10,000	> 10,000	> 10,000
CB1, CB2	> 10,000	> 10,000	> 10,000
DOR, KOR, MOR	> 10,000	> 10,000	> 10,000
H1-H4	> 10,000	> 10,000	> 10,000
Muscarinic receptors	> 10,000	> 10,000	> 10,000
NMDA	> 10,000	> 10,000	> 10,000
NOP	> 10,000	> 10,000	> 10,000
PBR	> 10,000	> 10,000	> 10,000
Sigma 2	> 10,000	> 10,000	> 10,000
Sigma1R	520	> 10,000	> 10,000

### Radiochemistry

**Fig. 2:**
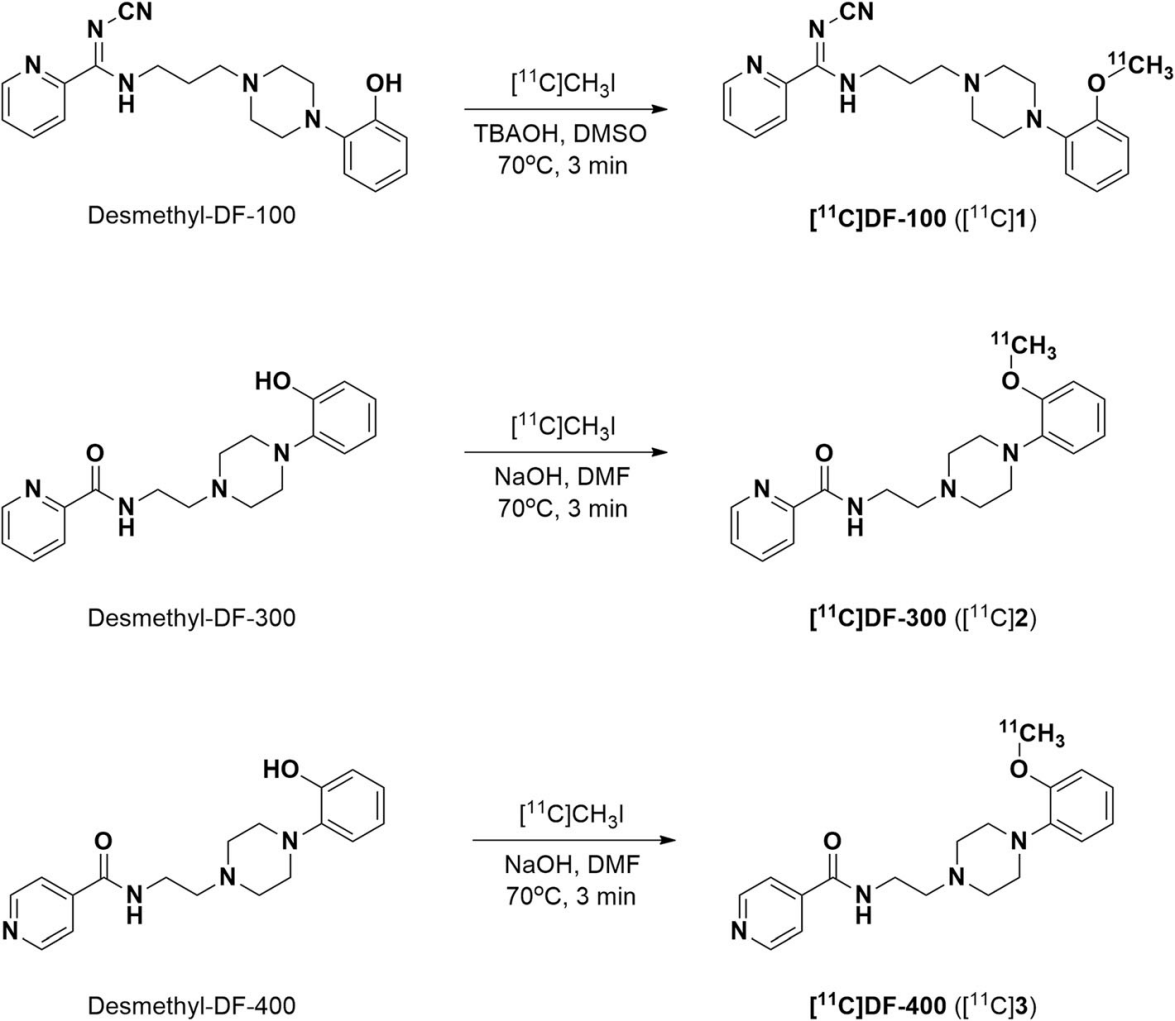
Chemical structures and radiochemical syntheses of the candidate 5-HT_1A_ radioligands: [^11^C]**1**, [^11^C]**2**, and [^11^C]**3**

Carbon-11 labelled compounds **1**–**3** were efficiently prepared by ^11^C-*O*-methylation of the corresponding phenolic precursor with [^11^C]methyl iodide ([^11^C]CH_3_I) as a methylating agent (Fig. 2). Given the susceptibility to in vivo metabolism at the methoxy site, we anticipated that ^11^C-*O*-methylation may prevent generation of brain-penetrating radiometabolites and thereby would not interfere with subsequent PET image quantification. Radioligands [^11^C]**1**–**3** were produced in a non-decay corrected RCY of 7–9% (relative [^11^C]CO_2_ (∼37 GBq)) and > 99% RCP using the present conditions in a commercially available and fully-automated ^11^C-methylation synthesis apparatus (GE TracerLab FX2 C). The overall synthesis time was 37–39 min, and the A_m_ was 81–224 GBq/μmol at the end-of-synthesis.

### In vivo PET imaging in rats

**Fig. 3:**
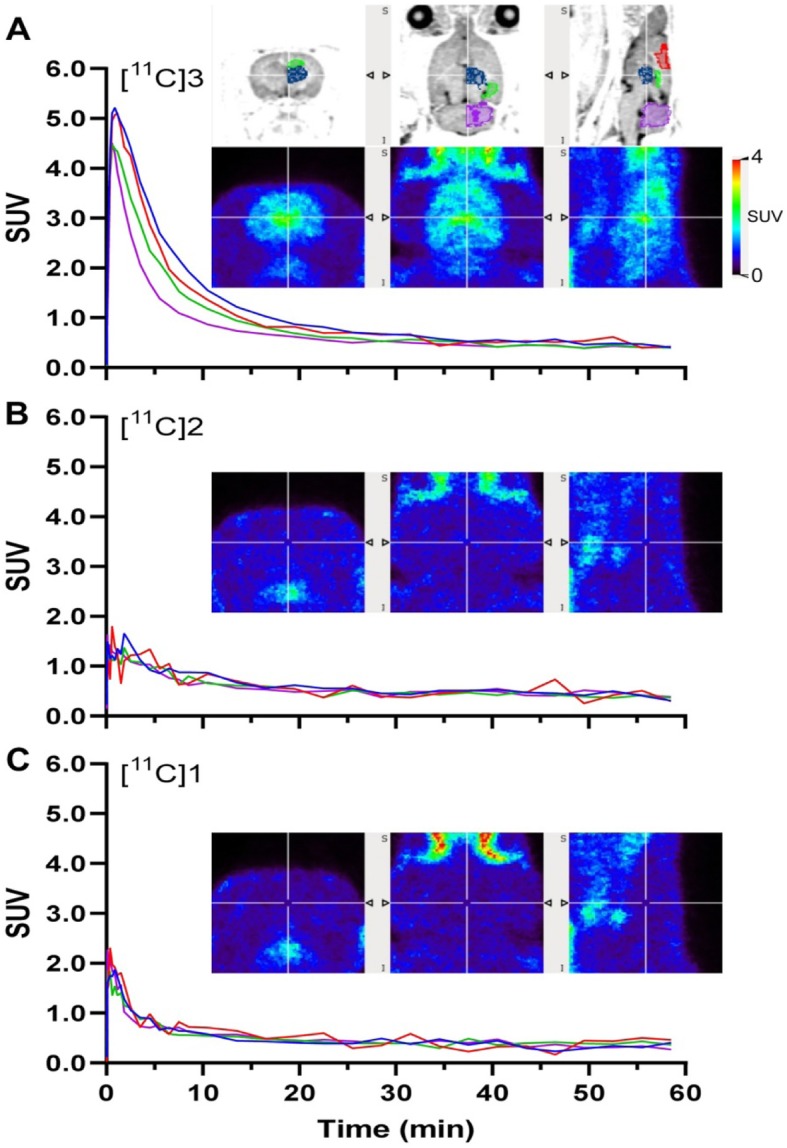
Uptake of [^11^C]**3** (**a**); [^11^C]**2** (**b**) and [^11^C]**1** (**c**) in rat brain. Shown are TACs averaged for left and right brain (A: *n* = 3; B and C: *n* = 1) in SUV and summed (0–60 min) PET images in coronal, transverse and sagittal planes, respectively, through the thalamus. The spatially co-registered MR images (2D fast spin echo) show left-half ROIs including thalamus (blue), anterior cingulate cortex (red), hippocampus (green) and cerebellum (magenta) for the corresponding color-coded TACs

Brain uptake of [^11^C]**1** and [^11^C]**2** was negligible (Fig. 3b and c). In contrast, [^11^C]**3** exhibited significant brain uptake demonstrating an early peak SUV of 4–5 at ~ 1 min following radiotracer injection (Fig. 3a). The washout of [^11^C]**3** was fast and the regional distribution and retention was different from that expected for 5-HT_1A_ binding areas based on previously reported neuroimaging studies (Wooten et al. 2011; Saigal et al. 2013). Inability of [^11^C]**1** and [^11^C]**2** to enter the brain may be attributed to structural modifications that influence efflux transporters and/or metabolizing enzymes (Pike 2009). [^11^C]**3** binding was observed in the following rank order: thalamus > prefrontal cortex (medial prefrontal, orbital frontal and anterior cingulate) > striatum > hippocampus and amygdala > occipital cortex > cerebellum (Fig. 3a; see Supplementary Figure 1 for regional BP values estimated with cerebellum as the reference region, see below for discussion).

**Fig. 4:**
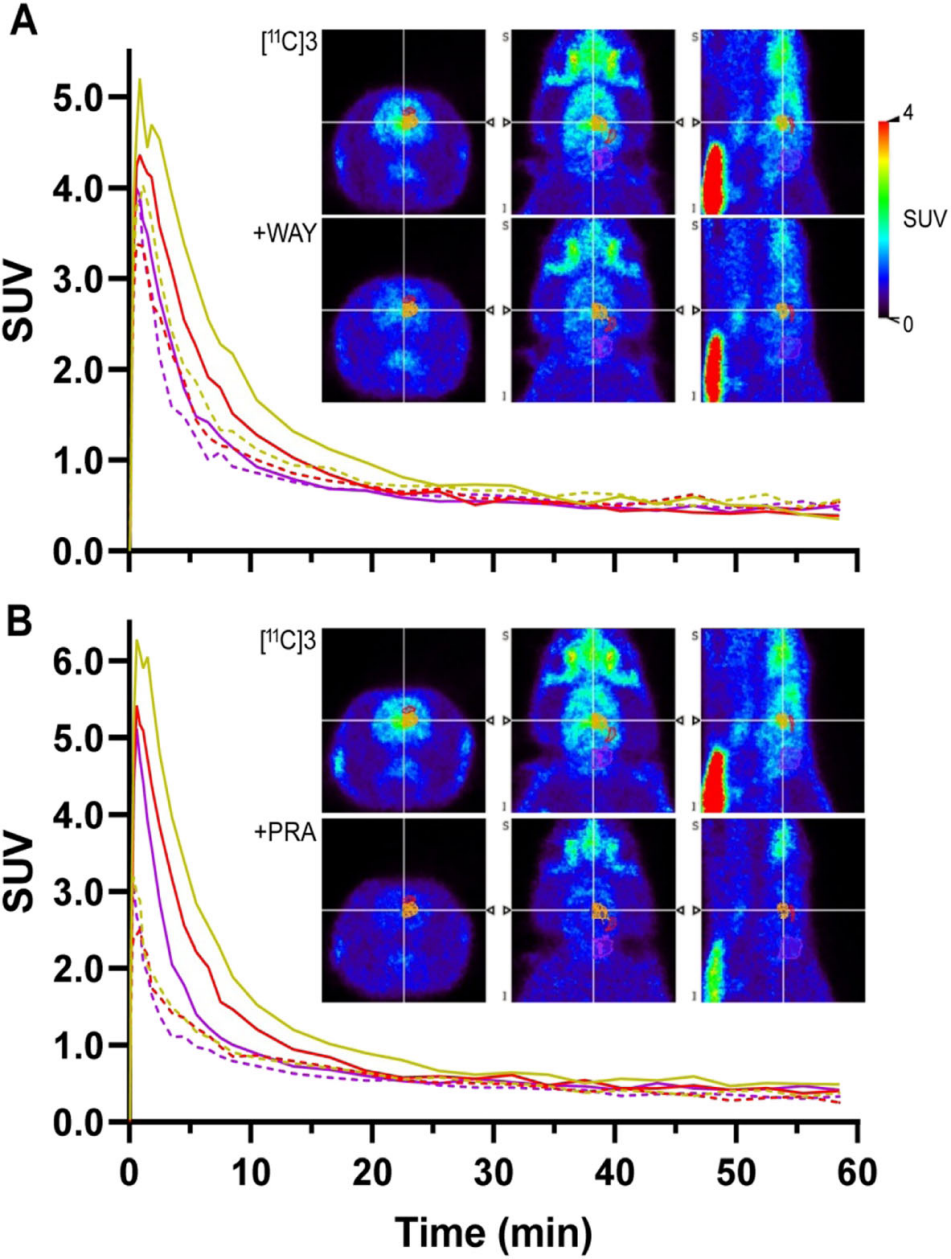
Blocking of the uptake of [^11^C]**3** in rat brain by WAY-100635 (**a**) and prazosin (**b**). Shown are TACs, averaged for left and right brain, (*n* = 1; solid: baseline; dashed: blocking) in SUV and summed (0–60 min) PET images in coronal, transverse and sagittal planes, respectively, through the thalamus at baseline and under blocking conditions. The three depicted left-half ROIs include thalamus (orange), hippocampus (red) and cerebellum (magenta) for the corresponding color-coded TACs

We further examined the in vivo specificity of [^11^C]**3** for 5-HT_1A_ by conducting a blocking study with WAY100635 (2 mg/kg; IV) that was administered 20 min prior to bolus injection of [^11^C]**3**. Comparison of TACs derived from baseline and blocking studies revealed decreased uptake of [^11^C]3 in all ROIs (Fig. 4a), including cerebellum, where the TAC showed a slightly faster descending phase but with the peak unchanged, suggesting the presence of some specific binding in this imperfect reference region but also that WAY100635 pretreatment did not change tracer delivery to brain. Whole brain BP was estimated to be decreased by 54%, including decreases in hippocampus (57%), anterior cingulate cortex (59%), thalamus (52%), and striatum (39%). Binding in the saliva gland was also decreased by 48%.

Given that thalamus has low 5-HT_1A_ receptor concentration, [^11^C]**3** binding to thalamus may represent an off-target binding site for the tracer. In fact, prior in vitro autoradiography studies conducted on rat brain tissue that employed both tritiated and iodinated α_1_-adrenergic antagonist based radioligands revealed high density of α_1_-adrenergic receptors in the thalamus (Jones et al. 1985; Unnerstall et al. 1985). This suggests the presence of significant off-target binding of [^11^C]**3** to α_1_-adrenergic receptors in thalamus which we further investigated by pre-treatment blocking studies. Pre-treatment with a selective α_1_-adrenergic receptor antagonist, prazosin (2 mg/kg; IV) 20 min prior to radiotracer injection markedly inhibited binding of [^11^C]**3** throughout the brain (Fig. 4b), with regional brain TACs all reduced to nearly overlap with that of cerebellum. Notably, the peak of the TACs was also markedly decreased, suggesting that prazosin, as a vasodilator, might have greatly influenced cerebral blood flow and tracer delivery to the brain. Whole brain BP was estimated to be decreased by 63%, including decreases in thalamus (73%), anterior cingulate cortex (69%), hippocampus (56%), and striatum (34%). Binding in the saliva gland was decreased by 68%.

The cerebellum has been commonly used as the reference tissue for 5-HT_1A_ PET tracers (Saigal et al. 2013; Shrestha et al., 2014). Preliminary PET imaging studies herein demonstrate lowest uptake of [^11^C]**3** in the cerebellum compared to other brain regions evaluated. To that end, the averaged TAC of left and right cerebellum was used as the reference region for the SRTM of Lammertsma and Hume (1996) to derive regional brain BP values. Nevertheless, it is important to note that BP values at baseline and the percentages of blocking might be somewhat underestimated given the small but displaceable binding in the cerebellum. Another limitation of the current investigation is that in vivo metabolite analyses of plasma and brain could not be justified ethically in light of the data. We therefore speculate that non-specific binding revealed in blocking studies may be attributed to non-specific binding of the parent compound to cellular components or accumulation of metabolite, and will be evaluated in future work should promising derivatives of [^11^C]**3** be identified.

Previous studies have reported cross affinity of 5-HT_1A_ ligands for α_1_-adrenergic receptors (Heimbold et al., 2002; Al Hussainy et al., 2011; Shrestha et al., 2014). [*carbonyl*-^11^C]WAY100635 (K_i_ = 2.2 nM), the most extensively studied 5-HT_1A_ antagonist PET radiotracer also demonstrates nanomolar affinity for α_1_-adrenergic receptors (K_i_ = 16.4 nM) (Chemel et al. 2006). While WAY100635 displays ~ 10-fold selectivity for 5-HT_1A_ receptors over α_1_-adrenergic receptors, in vivo cross selectivity of [*carbonyl*-^11^C] WAY100635 with α_1_-adrenergic receptors has not been reported. In the current study, in vitro data indicate ~ 4-fold selectivity of compound **3** to 5-HT_1A_ receptors over α_1_-adrenergic receptors which may be insufficient to establish in vivo selectivity for 5-HT_1A_ receptors in the imaging studies. The current results underscore the challenge posed by similarity between the transmembrane domains of 5-HT_1A_ receptors and α_1_-adrenergic receptor subtypes (Ngo et al., 2013). Taken together, development of ligands with considerably greater fold in vitro selectivity (~ 10 or preferably higher) for the 5-HT_1A_ receptors may be beneficial to avoid PET signal interference from binding to α_1_-adrenergic receptors.

## Conclusion

In conclusion, a high yielding radiosynthesis method to produce [^11^C]**3**, a potential 5-HT_1A_ antagonist-based radiotracer was achieved. Compound **3** exhibited approximately 4-fold selectivity to 5-HT_1A_ receptors over α_1_-adrenergic receptors. In vivo imaging experiments, however, demonstrated significant off-target binding, particularly in thalamus, which is attributed to α_1_-adrenergic receptors based on our blocking studies. Overall, results suggest that apart from thalamic binding, regional distribution of [^11^C]**3** corresponds to that of 5-HT_1A_ receptors. But the off-target binding limits the use of this radiotracer for quantification of 5-HT_1A_ receptors. Nevertheless, results reported herein contribute towards academic knowledge in this field that may support future radiotracer development efforts. Compound **3** represents a promising *O*-methylated lead candidate which if subjected to structural alterations, may either lead to improved selectivity for 5-HT_1A_ receptors or may assist in the development of the first PET radioligand for α_1_-adrenergic receptors.

## Supplementary information

**Additional file 1.**

## Data Availability

All data are included in the manuscript and supplementary information files.

## Abbreviations

α_1_: Alpha-1
Am: Molar Activity
BBB: Blood-Brain Barrier
BP: Binding Potential
FBKP: Filtered Back Projection.
FOV: Field of View
HPLC: High Performance Liquid Chromatography
8-OH-DPAT: 8-hydroxy-2-(di- n-propilamino)tetralin
NIMH: National Institute of Mental Health
PET: Positron Emission Tomography
PDSP: Psychoactive Drug Screening Program
ROIs: Regions of Interest
5-HT: Serotonin
SRTM: Simplified Reference Tissue Model
SUVs: Standardized Uptake Values
TACs: Time Activity Curves
UV: Ultraviolet
