# Supplementary material for: Synthesis, in vitro and in vivo evaluation of 11C-O-methylated arylpiperazines as potential serotonin 1A (5-HT1A) receptor antagonist radiotracers
Source: EJNMMI Radiopharm Chem. 2020 May 19;5:13. doi: 10.1186/s41181-020-00096-8 (PMC7237647; doi:10.1186/s41181-020-00096-8)

**Supporting Information**

**Synthesis, In Vitro and In Vivo Evaluation of ^11^C-*O*-Methylated Arylpiperazines as Potential Serotonin 1A (5-HT_1A_) Receptor Antagonist Radiotracers**

Vidya Narayanaswami^1^, Junchao Tong^1^, Ferdinando Fiorino^2^, Beatrice Severino^2^,

Rosa Sparaco^2^, Elisa Magli^2^, Flavia Giordano^2^, Peter M. Bloomfield^1^, Jaya Prabhakaran^3^,

J. John Mann^3,4^, Neil Vasdev^1,5^, Kenneth Dahl^1*^, and J.S. Dileep Kumar^3*^

^1^Azrieli Centre for Neuro-Radiochemistry, Research Imaging Centre & Preclinical Imaging, Centre for Addiction and Mental Health, Toronto, Ontario, M5T-1R8, Canada

^2^Department of Pharmacy, University of Naples, Via D. Montesano, 49, Naples 8013, Italy

^3^Molecular Imaging and Neuropathology Division, New York State Psychiatric Institute, New York, USA

^4^Department of Psychiatry, Columbia University Medical Center, New York, USA

^5^Department of Psychiatry, University of Toronto, Toronto, Ontario, M5T-1R8, Canada

* Corresponding authors:

[KennethF.Dahl@gmail.com](mailto:KennethF.Dahl@gmail.com);

[Dileep.Kumar@nyspi.columbia.edu](mailto:Dileep.Kumar@nyspi.columbia.edu)

***Chemical Synthesis of Radiolabeling Precursors***

***Synthesis of Desmethyl-DF-100***


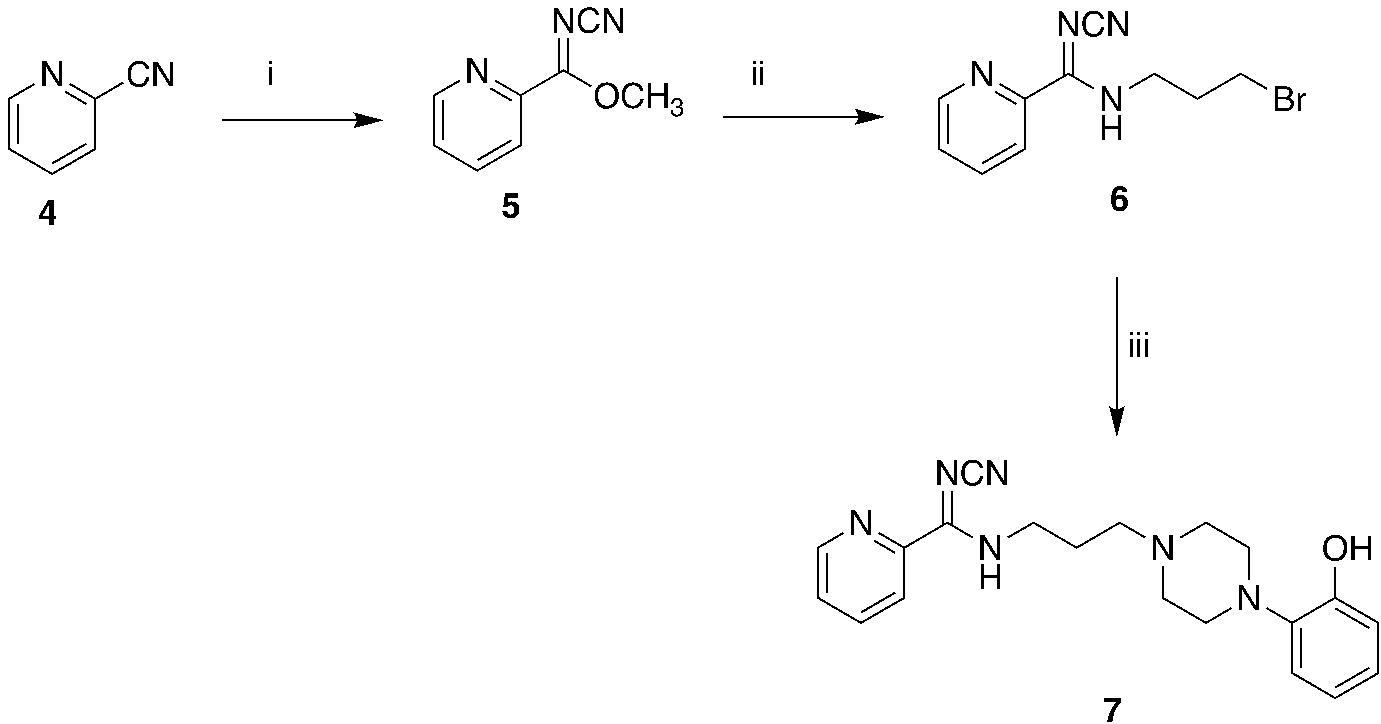


**Scheme 1.** Reagents and conditions: (i) CH_3_ONa, H_2_NCN, anhydrous MeOH; (ii) Br(CH_2_)_3_NH_2_∙HBr, TEA, anhydrous MeOH; (iii) 2-hydroxyphenyl-piperazine, K_2_CO_3_, NaI, CH_3_CN, 70 ^o^C, 4 h.

*Methyl N-cyano-2-pyridinecarboximidate* ***(5)***

A mixture of 2-cyanopyridine (**4**) (10 g, 0.096 mol) and NaOMe (0.26 g, 0.0048 mol) in anhydrous methanol (180 mL) was stirred overnight at room temperature. AcOH (0.32 g, 0.0053 mol) was then added with stirring to neutralize the reaction and the solution mixture was evaporated *in vacuum*. Diethyl ether (150 mL) was added to the residue and the resultant precipitate was filtered off. The filtrate was evaporated to give crude methyl-2-pyridinecarboximidate (7.0 g) as an oil. Crude compound was then added to a mixture of NH_2_CN (4.33 g, 0.103 mol), NaH_2_PO_4_· 2H_2_O (32.14 g) and Na_2_HPO_4_ (7.30 g) in water (55 mL). After vigorous stirring for 4 h at room temperature, the reaction mixture was extracted several times with CH_2_Cl_2_. The combined organic layers were dried on anhydrous Na_2_SO_4_ and concentrated in *vacuo* to yield crude methyl-*N*-cyano-2-pyridinecarboximidate (**5**) (8.4 g), which was used directly in the following reaction.

**5**: ^1^H-NMR (400 MHz, CDCl_3_) δ: 4.16 (s, 3H); 7.63 (dd, 1H, *J*= 9.4, 7.3 Hz); 7.94 (d, 1H, *J=3.4* Hz); 7.98 (dd, 1H, *J*= 7.3, 2.4 Hz); 8.83 (ddd, 1H, *J*= 9.4, 3.4, 2.4 Hz). ^13^C-NMR (100 MHz, CDCl_3_) δ: 46.35; 116.90; 123.64; 126.80; 136.25; 148.13; 149.15, 153.10.

*N-(3-bromopropyl)-N'-cyanopicolinamidine* ***(6)***

To a solution of methyl *N*-cyano-2-pyridinecarboximidate (**5**) (8 g, 0.050 mol) in anhydrous MeOH (120 mL), 3-bromopropylamine∙HBr (11.93 g, 0.055 mol) and triethylamine (5.56 g, 0.055 mol) were successively added and the reaction mixture was stirred at room temperature for 18 h. After evaporation, the residue was dissolved in CHCl_3_. The solution was washed with water, dried on anhydrous Na_2_SO_4_ and concentrated in *vacuo*. The residue was purified by column chromatography (diethyl ether/ ethanol 9:1 (v/v)). The combined and evaporated product fractions were crystallized from diethyl ether /hexane, yielding 8.4 g (63 %) of the desired product as a brown solid.

**6**: MP: 73-74°C; ^1^H-NMR (400 MHz, CDCl_3_) δ: 2.15 (qt, 2H, *J*=6.2 Hz); 2.75 (t, 2H, *J*=6.2 Hz); 3.83 (q, 2H, *J*=6.2 Hz); 7.47 (t, 1H, *J*=7.3 Hz); 7.86 (t, 1H, *J*=7.3 Hz); 8.5 (d, 1H, *J*= 7.3 Hz); 8.64 (d, 1H, *J*=7.3 Hz); 9.62 (s, 1H). ^13^C-NMR (100 MHz, CDCl_3_) δ: 27.33; 38.70; 53.91; 117.20; 122.54; 125.66; 135.10; 148.23; 150.70, 154.20.

*N'-Cyano-N-(3-(4-(2-hydroxyphenyl)piperazin-1-yl)propyl)picolinamidine* ***(Desmethyl-DF-100) (7)***

A mixture of *N*-(3-bromopropyl)-*N*'-cyanopicolinamidine (**6**) (0.006 mol), and NaI (0.009 mol) in acetonitrile was stirred under reflux for 30 min. Then 2-hydroxyphenyl-piperazine (0.06 mol) and anhydrous K_2_CO_3_ (0.009 mol) were added. The reaction mixture was stirred under reflux for 4 h. After cooling, the mixture was filtered, concentrated to dryness and the residue was dissolved in water (50 mL). The solution was extracted several times with CH_2_Cl_2_. The combined organic layers were dried on anhydrous Na_2_SO_4_ and the solvent removed under vacuum. The crude mixture was purified by silica gel column chromatography using diethyl ether/methanol 8:2 (v/v) as eluent. The crude product was recrystallized from diethyl ether in 24% yield.

**7**: MP: 137-138 ^o^C ^1^H-NMR (500 MHz, DMSO-d6) δ: 1.78 (m, 2H, *J=6.9* Hz), 2.43 (t, 2H, N^1^-CH_2_, *J=5.1* Hz); 2.51 (bs, 4H, 2CH_2_ pip.); 2.91 (bs, 4H, 2CH_2_ pip.); 3.53 (bs, 2H, N-CH_2_); 6.75-6.81 (m, 4H, Ar-OH); 7.63 (t, 1H, *J=7.3* Hz); 8.02 (t, 1H, *J=7.7* Hz); 8.68 (d, 1H, *J=8.0* Hz); 8.86 (d, 1H, *J=4.4* Hz); 9.64 (s, 1H); ESI-MS: 365.29 [M+H]^+^; 387.28 [M+Na]^+^.

***Synthesis of Desmethyl-DF-300 and Desmethyl-DF-400***


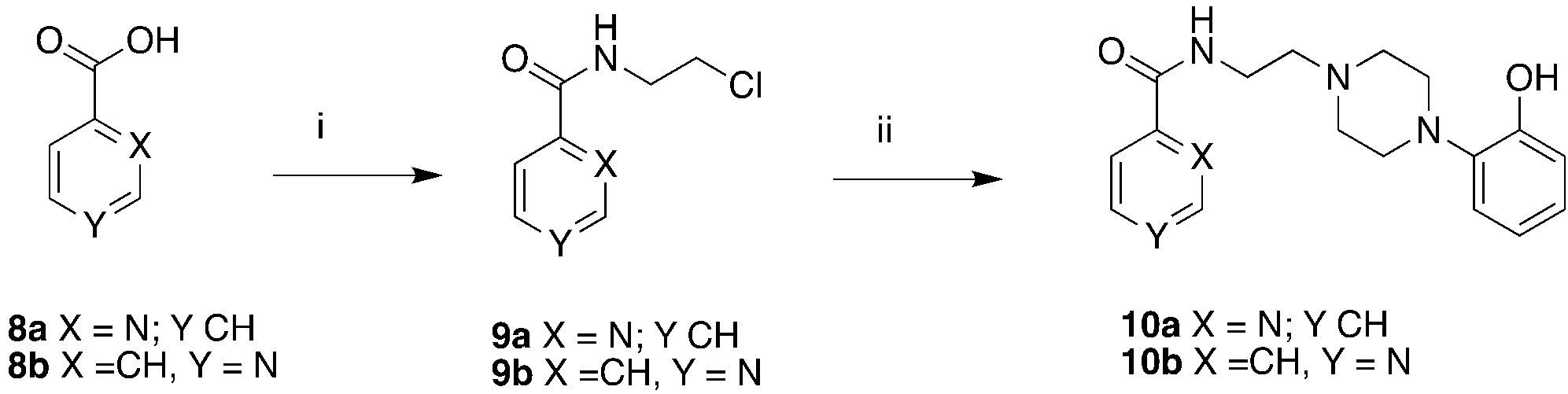


**Scheme 2.** Reagents and conditions: (i) Cl(CH_2_)_2_NH_2_·HCl, DCC, HOBt, TEA, CH_3_CN, r.t., 24h; (ii) 2-hydroxyphenyl-piperazine, K_2_CO_3_, NaI, CH_3_CN, 70°C, 24 h.

*General procedure for the synthesis of the N-(2-chloroethyl)picolinamide* **(9a)** *and N-(2-chloroethyl)isonicotinamide* ***(9b)***

*N,N'*-Dicyclohexylcarbodiimide (DCC, 9.28 g, 0.045 mol) and hydroxybenzotriazole (HOBt, 6.08 g, 0.045 mol) were added to a mixture of picolinic acid (**8a**) (5.00 g, 0.041 mol) or isonicotinic acid (**8b**) (5.00 g, 0.041 mol) in acetonitrile (50 mL) and the reaction was stirred for 1 h at 0^o^C. Then, triethylamine (TEA, 4.55 g, 0.045 mol) and the appropriate 2-chloroethanamine hydrochloride (4.75g, 0.041 mol) or 3-chloropropan-1-amine hydrochloride (5.33 g, 0.041 mol) was added to the reaction mixture and the resulting solution was stirred for 24 h at room temperature. The mixture was then cooled to 0°C in order to precipitate *N, N*′-dicyclohexylurea (DCU) that was filtered-off. The filtrate was evaporated and dissolved in CH_2_Cl_2_, the residue was washed with NaHCO_3_, water and brine. The combined organic layers were dried on anhydrous Na_2_SO_4_ and concentrated in *vacuo*. The crude *N*-(2-chloroethyl) derivatives **9a** or **9b** were purified by column chromatography (dichloromethane/methanol 9:1 (v/v)), yielding *N*-(2-chloroethyl)picolinamide (**9a**, 6.17 g, 82%), MP: 80-81 ^o^C;^1^H-NMR (400 MHz, CDCl_3_) δ: 3.61 (q, 2H, -NH-CH_2_, *J=5.4* Hz); 3.82 (t, 2H, -CH_2_-Cl, *J=5.4* Hz); 7.40 (t, 1H, *J=7.0* Hz); 7.85 (t, 1H, *J=7.0* Hz); 8.19 (d, 1H, *J=7.6* Hz); 8.40 (bs, 1H, NH); 8.56 (d, 1H, *J=5.8* Hz), or N-(2-chloroethyl)isonicotinamide (**9b**, 6.71 g, 89%), MP: 97-98 ^o^C; ^1^H-NMR (400 MHz, CDCl_3_) δ: 3.60 (q, 2H, -NH-CH_2_, *J=5.4* Hz); 3.85 (t, 2H, -CH_2_-Cl, *J=5.4* Hz); 6.99 (bs, 1H, NH); 7.61 (d, 2H, *J=5.8* Hz); 8.74 (d, 2H, *J=5.8* Hz). ^13^C-NMR (100 MHz, CDCl_3_) δ: 36.54; 56.36; 121.12; 142.65; 150.84; 165.54.

*General procedure for the preparation of N-(2-(4-(2-hydroxyphenyl)piperazin-1-yl)ethyl)picolinamide* ***(Desmethyl-DF-300) (10a)*** *and N-(2-(4-(2-hydroxyphenyl)piperazin-1-yl)ethyl)isonicotinamide* ***(Desmethyl-DF-400) (10b)***

A mixture of *N*-(2-chloroethyl)picolinamide (**9a**, 0.500 g, 2.71 mmol) or N-(2-chloroethyl)isonicotinamide (**9b**, 0.500 g, 2.52 mmol) and NaI (1.1 equiv.) in acetonitrile (30 mL) was stirred under reflux for 30 min. Then 2-hydroxyphenyl-piperazine (1.0 equiv.) and anhydrous K_2_CO_3_ (1.1 equiv.) were added. The reaction mixture was stirred under reflux for 24 h. After cooling to room temperature, the mixture was filtered, concentrated to dryness and the residue was dissolved in dichloromethane (20 mL), then washed with NaHCO_3_, water and brine. The combined organic layers were dried on anhydrous Na_2_SO_4_ and the solvent removed under *vacuum*. The crude mixture was purified by silica gel column chromatography using dichloromethane/methanol 9.5:0.5 (v/v) or ethyl acetate/methanol 9:1 (v/v) as eluent. The crude products were crystallized from diethyl ether, affording final compounds **10a** and **10b**.

*N-(2-(4-(2-hydroxyphenyl)piperazin-1-yl)ethyl)picolinamide* ***(10a)***: Yield: 38%; MP 112-113 ^o^C; ^1^H-NMR (400 MHz, DMSO-d_6_) δ: 2.47 (t, 2H, -CH_2_-N^1^, *J*=5.4 Hz); 2.52 (t, 4H, 2CH_2_ pip., *J*=4.7 Hz); 2.91 (t, 4H, 2CH_2_ pip., *J*=4.7 Hz); 3.42 (q, 2H, -NH-CH_2_, *J*=5.4 Hz); 6.69-6.82 (m, 4H, Ar-OH); 7.57 (t, 1H, J=7.0); 7.97 (t, 1H, *J*=7.0); 8.01 (d, 1H, *J*=7.6); 8.62 (d, 1H, *J*=5.8); 8.89 (bs, 1H, NH); ESI-MS: 327.26 [M+H]^+^, 349.21[M+Na]^+^.

*N-(2-(4-(2-hydroxyphenyl)piperazin-1-yl)ethyl)isonicotinamide* ***(10b)***: Yield: 45%; MP 105-106 ^o^C; ^1^H-NMR (400 MHz, DMSO-d_6_) δ: 2.50 (t, 2H, -CH_2_-N^1^, *J=5.4* Hz); 2.56 (bs, 4H, 2CH_2_ pip., *J=4.7* Hz); 2.90 (bs, 4H, 2CH_2_ pip., *J=4.7* Hz); 3.49 (q, 2H, -NH-CH_2_, *J=5.4* Hz); 6.69-6.82 (m, 4H,Ar-OH); 7.71 (d, 2H, *J=5.8* Hz); 8.69 (d, 2H, *J=5.8* Hz); 8.89 (bs, 1H, NH).

**Supplementary Figure 1:** Regional brain binding potentials (BP) of [^11^C]**3 (**n = 3) derived from the average values of left and right half brain (mean ± SEM). The averaged time-activity curve of left and right cerebellum was used as the reference region for the simplified reference tissue model (SRTM) of Lammertsma (1996). It should be noted that the BP values might be somewhat underestimated, given the presence of small but displaceable binding in cerebellum (see Figure 4).


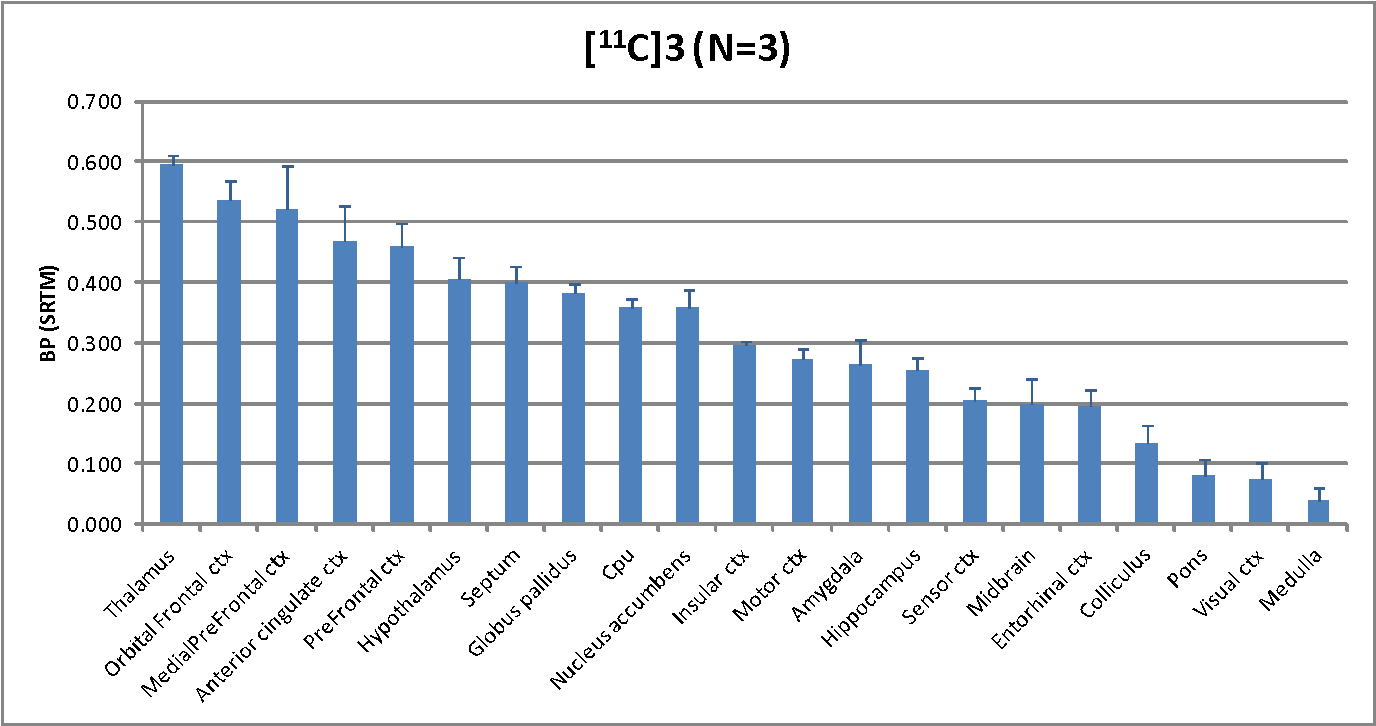

Supplement: Supplementary file 1 — Additional file 1. [file 41181_2020_96_MOESM1_ESM.doc]
